# Multimeric fusion single‐chain variable fragments as potential novel high‐capacity ligands

**DOI:** 10.1002/2211-5463.12789

**Published:** 2020-03-03

**Authors:** Laila I. Sakhnini, Anja K. Pedersen, Maria B. Dainiak, Leif Bülow

**Affiliations:** ^1^ Global Research Technologies Novo Nordisk A/S Copenhagen Denmark; ^2^ Division of Pure and Applied Biochemistry Lund University Lund Sweden; ^3^ Chemistry, Manufacturing and Control Novo Nordisk A/S Copenhagen Denmark

**Keywords:** affinity chromatography, binding capacity, peptide linker, recombinant fusion protein, single‐chain variable fragment

## Abstract

In basic and applied biotechnology, design of affinity ligands has become essential for high‐capacity applications such as affinity‐based downstream processes for therapeutic molecules. Here, we established a proof‐of‐concept for the use of multimeric fusion single‐chain variable fragment (scFvs) as high‐capacity ligands in affinity adsorbents. Mono‐ and di/tri‐scFvs separated by Pro‐rich negatively charged linkers were designed, produced, and immobilized to 6% cross‐linked agarose beads. Frontal binding experiments with a target protein of 50 kDa resulted in up to 20 mg·mL^−1^ and 82% in dynamic binding capacity and utilization yield, respectively, at 100% breakthrough. The utilization of the binding sites was impacted by the ligand format and ligand density, rather than limitation in pore size of adsorbent as previously suggested. Overall, we demonstrated that multimeric fusion scFvs can successfully be developed and used as high‐capacity ligands in affinity adsorbents, enabling lean process design and alignment with process specifications.

## Graphical abstract

In this study, a proof‐of‐concept was established for the use of multimeric fusion single‐chain variable fragment (scFvs) as high‐capacity ligands in affinity adsorbents. Mono‐ and di/tri‐scFvs, separated Pro/Glu repeats, were successfully produced and immobilised to 6% cross‐linked agarose via C‐terminal Cys. Frontal affinity chromatography experiments resulted in binding capacity and utilization yield of up to 20 mg·mL^−1^ and 82%, respectively, at 100% breakthrough.

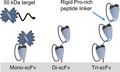

**Abbreviations**
CHO: Chinese hamster ovary
CV: column volume
DBC: dynamic binding capacity
Di: dimeric
mAb: monoclonal antibody
Mono: monomeric
MWCO: molecular weight cutoff
scFv: single‐chain variable fragment
TCEP: Tris (2‐carboxyethyl) phosphine
*T*_m_: temperature of midpoint of thermal unfolding
Tri: trimeric
*V*_H_: variable heavy chain
*V*_L_: variable light chain

Affinity chromatography is a commonly used technique for purification of specific target proteins. It has shown to be ideal for direct capture of low‐expressing proteins from crude starting materials 1. However, it can be challenging to find suitable affinity adsorbents for certain proteins as the commercial market is mostly limited to affinity adsorbents for purification of affinity‐tagged target proteins 2, 3, 4 and monoclonal antibodies (mAbs) 5, 6, 7, 8, 9, 10. Nevertheless, if there is an antibody available against a target protein of interest, an immunoaffinity adsorbent can be developed by immobilization of the antibody to a chemically activated resin, such as cross‐linked agarose, cellulose, or synthetic polymers 11.

Important characteristics to consider when developing affinity adsorbents are chemical stability, resin lifetime, and binding capacity. In particular, the binding capacity has a great impact on manufacturing costs and productivity 12. At the manufacturing scale, affinity adsorbents with low binding capacity can require large‐sized columns with up to 2 m in diameter for increasing productivity. The drawbacks of these large columns include (a) additional costs due to large volumes of adsorbent, buffers, and consumables needed; (b) design of a facility to accommodate the space needed for the unit operation; and (c) pressure drop and unpredictable fluid distribution caused by issues in the column packing, and compression of affinity adsorbent 13.

The current trend in the preparation of immunoaffinity adsorbents has been to immobilize smaller fragments of antibodies to get higher dynamic binding capacities (DBC), such as the single‐chain variable fragment (scFv) 14 and the heavy variable domains of camelid antibodies (nanobodies) 15. The commercial immunoaffinity adsorbents KappaSelect, LambdaSelect, and VIIISelect are three examples of nanobody‐based affinity adsorbents that provide a high binding capacity, typically between 8 and 20 mg·mL^−1^ 10, 16. Nevertheless, maximum binding capacity of these adsorbents can only be achieved via monolayer adsorption as they accommodate one antigenic binding site per ligand only. The concept of a multimeric affinity ligand may be an alternative to increase capacity further and it has shown to be successful for the bacterial‐derived protein A‐based hexameric ligand in MabSelect 17. However, a thorough literature search has returned no findings on the development of recombinant multimeric fusion antibody fragment‐based affinity ligands, despite the development of multimeric antibody‐based formats, such as diabodies and divalent scFvs, as emerging potential drugs and probes in the biopharmaceutical industry 18, 19.

The aim of this study was to establish a proof‐of‐concept for the use of multimeric fusion scFvs as high‐capacity affinity ligands. The approach consisted of the following steps: (a) development of high‐expressing dimeric (di‐) and trimeric (tri‐) scFv ligands by assessment of peptide linkers differing in rigidity and length, (b) site‐directed immobilization of the multimeric scFv ligands via thiol groups to chemically activated cross‐linked agarose beads, and (c) characterization of the developed affinity adsorbents by frontal affinity chromatography and adsorption isotherms. We report on the successful development of multimeric scFvs separated by negatively charged, rigid peptide linkers. We demonstrate that both monomeric (mono‐) and multimeric scFv‐based affinity adsorbents can result in close to fully accessible binding sites and substantially higher binding capacity than previously reported for immunoaffinity adsorbents 14.

## Results and Discussion

### Design of multimeric fusion scFvs

**Figure 1:**
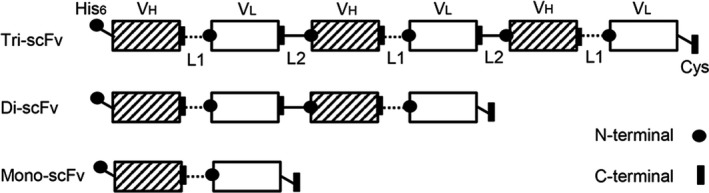
Schematic representation of the designed constructs encoding the multimeric scFv ligands. Antibody domain is indicated by *V*_H_ and *V*_L_. Linker is indicated by L1: flexible linker (G_4_S)_3_ and L2: rigid linkers (EP)*_k_*, (ESP)*_m_*, and (ESEP)*_n_*, where (*k* = 6 and 8), (*m* = 4 and 6), and [*n* = 3 and 4].

In order to develop a multimeric fusion protein, a suitable peptide linker has to be chosen. Peptide linkers can either be flexible, rigid, or cleavable depending on the desired characteristic 20. In the case of multimeric fusion scFvs, a linker that can impose spatial separation is crucial for preserving independent folding and functionality. For this purpose, rigid linkers, such as helical structures [e.g., (EAAAK)*_n_*, where *n* is the copy number] or Pro‐rich sequences [e.g., (XP)*_n_*, where X is any amino acid], have shown to be the desired choice 20, 21. Particularly, Pro‐rich sequences have shown to impose stiff and extended conformations and their length can be adjusted by the copy number 22. Hence, three different Pro‐rich linkers differing in length and rigidity were chosen to be investigated in terms of expression level and spatial separation: (EP)*_k_*, (ESP)*_m_*, and (ESEP)*_n_*, where (*k* = 6 and 8), (*m* = 4 and 6), and (*n* = 3 and 4) (Fig. 1). Glu and Ser were selected as they had previously been shown to increase protein solubility 23.

**Figure 2:**
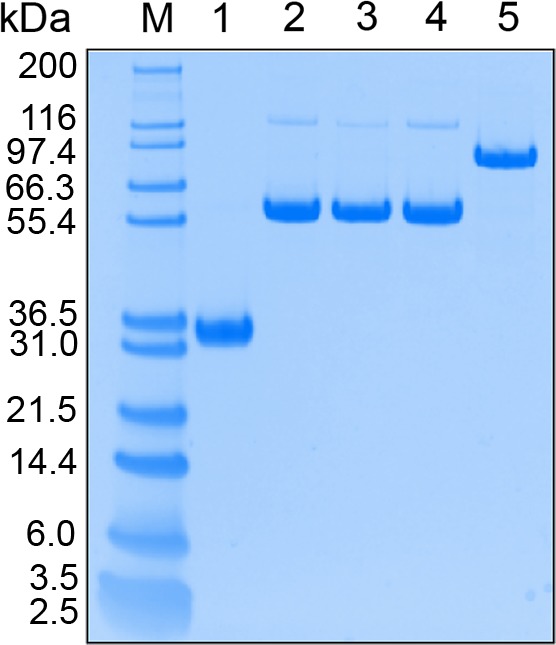
SDS/PAGE gel of purified affinity ligands with selected linkers; lane (M), Molecular weight standard (Marker12); lane (1), mono‐scFv; lane (2), di‐scFv (EP)_8_; lane (3), di‐scFv (ESP)_6_; lane (4), di‐scFv (ESEP)_4_; and lane (5), tri‐scFv (EP)_8._

Produced di‐scFvs with linker length of 16‐18 resulted in substantially higher recovery relative to that of 12 residues (Fig. S1). Therefore, the longer linker length was selected. The effect of the linker rigidity on the expression level was not as pronounced as the effect of the linker length. However, di‐scFvs with the most rigid linker, (EP)_8_, seemed to result in slightly higher recovery relative to di‐scFvs with (ESP)_6_ and (ESEP)_4_ linkers. Based on these findings, the (EP)_8_ was selected for further development of a tri‐scFv. Produced mono‐, di‐, and tri‐scFv ligands were of high purity (Fig. 2), and their identities were verified by LC‐MS analyses (data not shown).

To investigate whether the linkers could impose spatial separation between the scFvs, the temperature of the midpoint of thermal unfolding (*T*_m_) was determined for the highest expressed ligands. Identical proteins that are separated by a linker should theoretically exhibit one unfolding transition at the same *T*_m_. All di‐ and tri‐scFvs exhibited one unfolding transition at a temperature close to the *T*_m_ of mono‐scFv (Table S1 and Fig. S2). This indicates that all of the three rigid linkers could separate the scFvs. This is essential for preserving the accessibility of the antigenic binding sites of the ligands.

### Characteristics of immunoaffinity adsorbents

**Figure 3:**
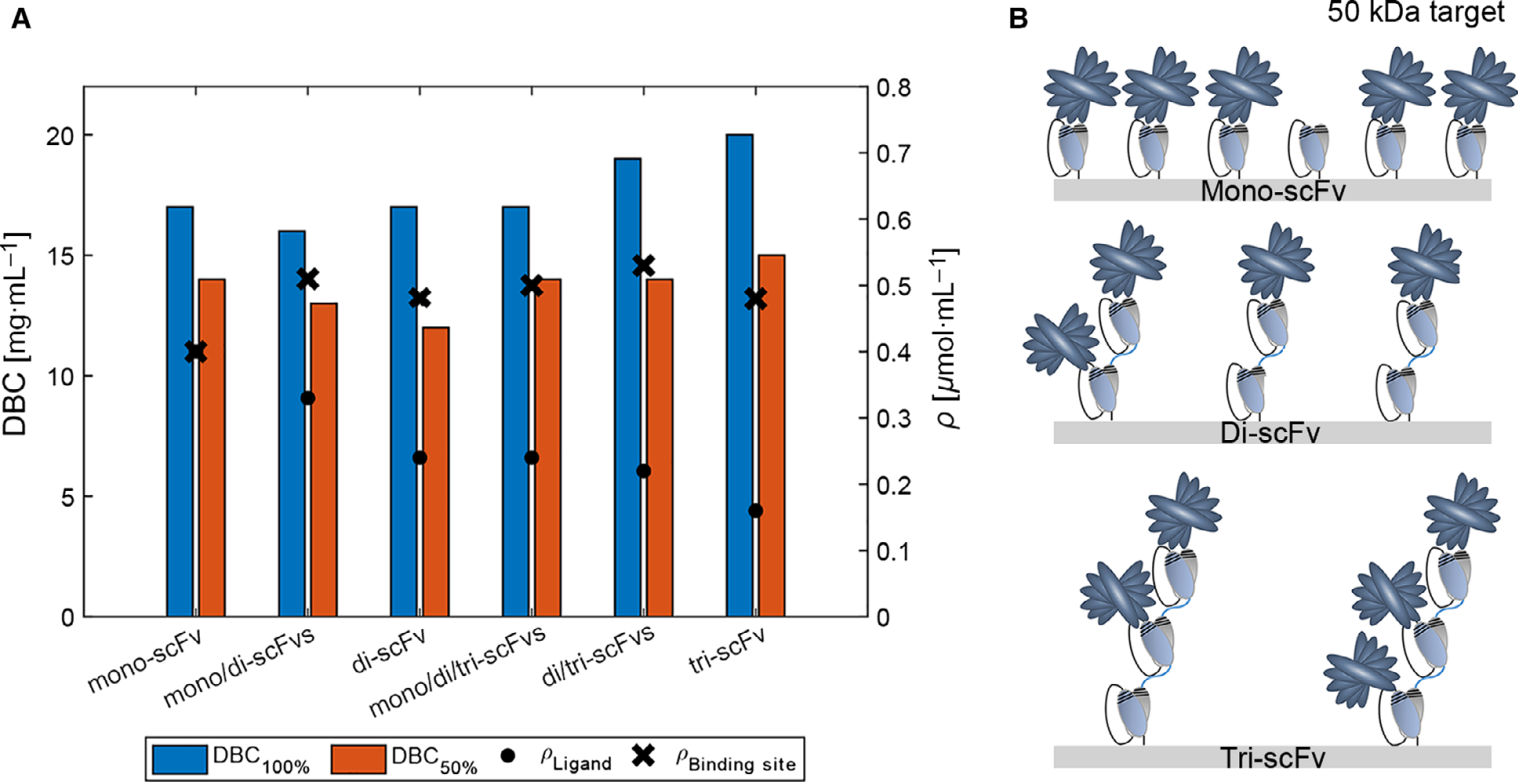
(A) A bar chart showing the DBC_100%_, DBC_50%_, and ρ_Ligand_ for the affinity resins with approximately equal ρ_Binding site_. (B) A schematic representation of how the mono/di/tri‐scFv ligands bound to target protein (50 kDa) may appear at 100% breakthrough.

Mono‐, di‐, and tri‐scFvs were successfully immobilized, both individually and in combination, via thiol‐directed chemistry to 6% cross‐linked agarose beads (sulfhydryl‐reactive resin; Divbio Science, Ulvenhout, Netherlands). In total, nine different immunoaffinity resins were developed and characterized in terms of immobilization yield, ligand density, binding capacity, and utilization yield. The ligand densities and immobilization yields ranged from 0.08 to 0.40 µmol·mL^−1^, and 80 to 97%, respectively (Table 1 and Fig. 3). No apparent effect of the linker rigidity was observed on the binding capacity as the obtained DBC_100%_ was approximately equal at the same ligand density (15–17 mg·mL^−1^ at 0.24–0.26 µmol·mL^−1^, see Table 1). Site‐directed mono‐scFv resulted in a DBC_100%_ and utilization yield of 17 mg·mL^−1^ and 82%, respectively, at a ligand density of 0.40 µmol·mL^−1^. This is a further improvement compared to the previously reported random‐oriented mono‐scFv with DBC_100%_ and utilization yield of 10 mg·mL^−1^ and 67%, respectively, at a ligand density of 0.30 µmol·mL^−1^ 14.

**Table 1:** The characteristics of the developed affinity resins: density of binding sites (ρ_Binding site_), ligand density (ρ_Ligand_), immobilization yield (θ), and DBC and utilization yield at 50% and 100% breakthrough, respectively.

Number	Affinity resin	ρ_Binding site_ (µmol·mL^−1^)	ρ_Ligand_ (µmol·mL^−1^)	θ (%)	DBC_50%_ (mg·mL^−1^)	Utilization yield_50%_ (%)	DBC_100%_ (mg·mL^−1^)	Utilization yield_100%_ (%)
1	Mono‐scFv	0.40	0.40	80	14	68	17	82
2	Di‐scFv (EP)_8_	0.16	0.08	97	6.0	74	6.8	85
3	Di‐scFv (EP)_8_	0.48	0.24	95	12	48	17	69
4	Di‐scFv (ESP)_6_	0.50	0.25	95	11	42	15	61
5	Di‐scFv (ESEP)_4_	0.52	0.26	96	11	41	15	56
6	Tri‐scFv (EP)_8_	0.48	0.16	91	15	63	20	82
7	Mono/Di‐scFv (EP)_8_	0.51	0.15/0.18	81/98	13	51	16	63
8	Di/Tri‐scFv (EP)_8_	0.53	0.10/0.11	90/97	14	53	19	71
9	Mono/Di/Tri‐scFv (EP)_8_	0.50	0.07/0.08/0.09	80/91/96	14	54	17	67

Increased density of di‐scFv from 0.08 to 0.24 µmol·mL^−1^ resulted in increased DBC_100%_ from 6.8 to 17 mg·mL^−1^ albeit decreased utilization yield by approximately 16%. Tri‐scFv resulted in binding capacity and utilization yield of 20 mg·mL^−1^ and 82%, respectively, thereby on par with the mono‐scFv. Highest utilization yield at 50% breakthrough was obtained for mono‐ and tri‐scFvs (63–68%) (Fig. 3A and Table 1). Furthermore, maximum utilization of the capacity can be achieved by recycling of FT in a continuous capture mode 24.

### Equilibrium binding capacity

Four of the developed affinity adsorbents were selected for batch adsorption experiments: mono‐scFv (0.40 µmol·mL^−1^), di‐scFv (0.08 and 0.24 µmol·mL^−1^), and tri‐scFv (0.16 µmol·mL^−1^). The obtained data at equilibrium were fitted to the Langmuir adsorption model (Fig. S3), which assumes monolayer adsorption. This was not expected as multilayer adsorption behavior was anticipated for the di‐ and tri‐scFvs ligands. A model for multilayer adsorption was tested, that is, the Brunauer–Emmett–Teller model; however, similarly shaped isotherms as for the Langmuir model were obtained (data not shown) 25. A summary of the estimations can be seen in Table 2. Overall, estimated *q*_m_ seem to be reasonable as they do not exceed the theoretical capacities. Highest *q*_m_ and utilization yield at equilibrium were obtained for tri‐scFv (22 mg·mL^−1^; 89%) and mono‐scFv (19 mg·mL^−1^; 92%). This suggests that the binding sites are close to fully accessible. In the case of the di‐scFv ligands, slightly lower *q*_m_ and utilization yields were obtained relative to the DBC_100%_ and utilization yields in Table 1.

**Table 2:** Maximum theoretical binding capacity, utilization yield, and maximum binding capacity at equilibrium (*q*_m_) of selected immunoaffinity resins.

Number	Affinity resin	ρ (µmol·mL^‐1^)	Theoretical capacity^a^ (mg·mL^‐1^)	$q_{m}^{b}$ (mg·mL^‐1^)	Utilization yield^b^ (%)
1	Mono‐scFv	0.40	21	19 (15–23)	92
2	Di‐scFv (EP)_8_	0.08	8.3	5.6 (3.4–7.8)	68
3	Di‐scFv (EP)_8_	0.24	25	14 (11–18)	58
4	Tri‐scFv (EP)_8_	0.16	25	22 (16–28)	89

a Maximum theoretical capacity calculated by ρ·N·M_target_

A 95% confidence interval is shown within the brackets

b Calculation based on mean *q*_m_.

### Capture of target protein at low titer

**Figure 4:**
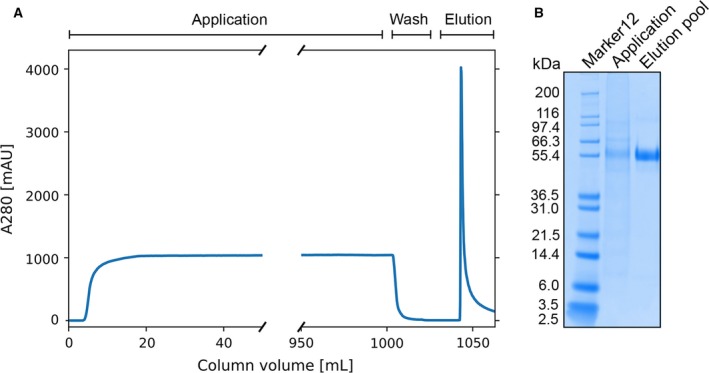
Capture test of a multimeric affinity resin, tri‐scFv affinity resin, with CHO supernatant with target protein X (< 10 µg·mL^−1^). (A) Experimentally obtained chromatogram; (blue curve) absorbance at 280 nm. (B) SDS/PAGE gel; lane (M), molecular weight standard (Marker12); lane (1), application (CHO supernatant with target protein X); and lane (2), elution pool (captured target protein X).

A multimeric scFv affinity adsorbent, tri‐scFv, was evaluated with Chinese hamster ovary (CHO) supernatant containing target protein X (< 10 µg·mL^−1^). Although 1 L of CHO supernatant was applied to the tri‐scFv column [column volume (CV) = 0.5 mL], the target protein could successfully be captured with sufficient purity (Fig. 4). This demonstrates that the multimeric adsorbent performs well at low titer.

### Future studies and potential applications

As the concept of multimeric fusion scFvs showed to be developable, functional, and on par with the mono‐scFv, a follow‐up study should investigate the potential benefit of higher order fusion scFvs and optimize the ligand density for maximization of the DBC. In addition, ligand leaching, regeneration procedure, and resin reusability should be assessed. Furthermore, the use of high‐capacity affinity ligands can be of particular interest in analytical applications such as biosensors and ELISA. A common bottleneck in these assays is low sensitivity, thereby making detection of low‐abundant molecules, for example, biomarkers, challenging. However, a way to improve the sensitivity is to increase the binding capacity in order to decrease the limit of detection 26, 27, 28. Thus, a possibility to improve the sensitivity of such assays could be to use high‐capacity multimeric fusion scFvs instead of conventional reagents.

## Concluding remarks

Overall, we demonstrated that multimeric fusion scFvs can successfully be developed and used as high‐capacity affinity ligands. The rigid linkers, (EP)_8_, (ESP)_6_, and (ESEP)_4_, successfully imposed separation, which is essential for preserving independent folding and facilitating accessible binding sites of the scFvs. Overall, site‐directed immobilization of the ligands to 6% cross‐linked agarose resins resulted in DBC_100%_ and utilization yields of up to 20 mg·mL^−1^ and 82%, respectively. Altogether, when binding to a target protein of 50 kDa, ligand format, site direction, and ligand density had a substantial effect on the binding capacity, rather than a limitation in pore size as previously suggested 14. As proof‐of‐concept was established for the suitability of multimeric scFvs as ligands in affinity adsorbents, future studies aim to optimize properties such as multimeric ligand format and ligand density for maximization of binding capacity, as well as evaluate the chemical stability and resin lifetime.

## Materials and methods

### Materials

Expi293™ Expression System Kit was obtained from Life Technologies (Carlsbad, CA, USA). Corning™ Disposable Vacuum Filter (1‐L scale, 0.22 µm) and materials for SDS/PAGE were obtained from Thermo Fisher Scientific (Waltham, MA, USA). Vivaspin 6 (MWCO: 10 kDa) was acquired from Sartorius Stedim Biotech (Göttingen, Germany). Ni Sepharose High Performance was obtained from GE Healthcare (Uppsala, Sweden). The sulfhydryl‐reactive resin was obtained from Divbio Science. The reagent utilized for the reduction of the C‐terminal Cys was Tris (2‐carboxyethyl) phosphine (TCEP) purchased from Thermo Fisher Scientific. Chemicals used for buffer preparation were purchased from Sigma‐Aldrich (St. Louis, MO, USA) unless otherwise stated.

### Construct generation

DNA sequences encoding V_H_‐(G_4_S)_3_‐V_L_ (scFv), scFv‐linker‐scFv, and scFv‐linker‐scFv‐linker‐scFv with N‐terminal His_6_ purification tags were designed based on the DNA sequence of a full‐length anti‐protein X antibody raised in mice. The linker was (EP)*_k_*, (ESP)*_m_*, and (ESEP)*_n_*, where (*k* = 6 and 8), (*m* = 4 and 6), and (*n* = 3 and 4). The DNA fragments were synthesized and cloned in the pTT5 vector by GeneArt (Thermo Fischer Scientific).

### Protein expression and purification

Plasmid DNA production and transient protein expression in mammalian HEK293 cells (0.5–1 L scale) were performed as previously described 29. Produced proteins were purified in a single step by immobilized metal ion affinity chromatography (IMAC) using Ni Sepharose High Performance column (16 mm × 5 cm; CV, 10 mL) with a linear flow velocity of 75 cm·h^−1^ (2.5 mL·min^−1^). Prior to column loading, 300 mm sodium chloride and 5 mm imidazole were added to the harvested protein supernatants (0.5–1.0 L). The running buffers were equilibration buffer (20 mm sodium phosphate, 0.5 m sodium chloride, 10 mm imidazole; pH 7.4) and elution buffer (20 mm sodium phosphate, 0.5 m sodium chloride, 0.5 m imidazole; pH 7.4). The scFv fragments were eluted by linear elution gradient (0–100% elution buffer for 10 CVs), and the collected elution fractions were pooled. Protein concentrations were estimated by NanoDrop™ 8000 Spectrophotometer (Thermo Fisher Scientific) at 280 nm (molar extinction coefficient 56160 m ^−1^·cm^−1^ at 280 nm).

### General analytical methods

The following standard analyses were performed according to standard methods described previously 30. Qualitative protein analysis was performed by SDS/PAGE. Protein identity verification was performed by LC‐MS. Temperature of midpoint of heat denaturation (*T*_m_) was determined by nano differential scanning fluorimetry for protein samples in triplicates at 0.5 mg·mL^−1^ in 50 mm sodium phosphate and 150 mm sodium chloride (pH 7.0).

### Preparation of affinity adsorbents and affinity chromatography

Site‐directed immobilization of the ligands via thiol groups to sulfhydryl‐reactive resin, and determination of immobilization yield (θ) and ligand density (ρ_Ligand_) were performed as previously described 14. In addition, the density of binding sites (ρ_Binding site_) was determined according to Eq. 1.

(1) $$\rho_{\text{Binding}\;\text{site}}=\sum_{i}^{n}N_{i}\cdot \rho_{\text{Ligand},i}$$

where *i* is the index number of the ligand and *N_i_* is the number of antigen‐binding sites of ligand *i*. The developed affinity adsorbents were packed into Tricorn 5/50 Columns (5 mm × 2.5 cm; CV = 0.5 mL) and evaluated in terms of dynamic binding capacity (DBC) and utilization yield by frontal affinity chromatography with pure target protein X (0.4 mg·mL^−1^) as previously described 14.

### Capture of target protein from CHO expression

Calcium chloride was added to a final concentration of 20 mm to 1 L of CHO mammalian cell harvest containing target protein X (< 10 μg·mL^−1^). Frontal affinity chromatography was carried out as previously described 14.

### Batch adsorption experiments

Batch adsorption experiments were performed according to the following. Six different start concentrations of protein X in equilibration buffer (10 mm l‐histidine, 100 mm sodium chloride, 25 mm calcium chloride; pH 6.0) were prepared at 0.5, 1.0, 2.0, 4.0, 6.0, and 8.0 mg·mL^−1^, respectively. Prior to experiments, affinity resins were washed at least three times with equilibration buffer. For each selected affinity resin, 0.10 mL of 1 : 1 mixture of solid/liquid resin slurry was added per well in a 96‐well plate for each concentration of target protein. 0.15 mL of protein target solution was added to each well. The 96‐well plate was incubated on a shaker at 4 °C for 6 h in order to reach equilibrium. After incubation, the plate was centrifuged at 1000 ***g*** for 2 min using the Heraeus Multifuge 3SR Plus (Thermo Fisher Scientific); thereafter, supernatants were analyzed by NanoDrop™ 8000 Spectrophotometer (Thermo Fisher Scientific). Experiments were performed in duplicates. Experimentally obtained data were fitted to the Langmuir isotherm by nonlinear regression deploying the least‐square‐sense fitting lsqcurvefit algorithm in MATLAB version R2019a (MathWorks, Natick, MA, USA). 95% confidence intervals were computed for the fitted parameters.

## Conflict of interest

The authors declare no conflict of interest.

## Author contributions

LIS conceived and designed the project, performed the experiments, and wrote the paper. All authors contributed to the interpretation of the data and the final version of the manuscript.

## Supporting information


**Table S1** **.** Temperature of mid‐point of heat denaturation (*T*_m_) of mono‐, di‐ and tri‐scFvs with selected linkers.
**Fig. S1**. Elution profiles from IMAC chromatography for multimeric ligands with different linker lengths. (A) di‐scFv ligands with (EP)_6_ and (EP)_8_, (B) di‐scFv ligands with (ESP)_4_ and (ESP)_6_, (C) di‐scFv ligands with (ESEP)_3_ and (ESEP)_4_, and (D) tri‐scFv ligand with (EP)_8_. Application corresponds to 0.5 L of HEK293 supernatant.
**Fig. S2**. Unfolding curves from nano differential scanning fluorimetry; (A) mono‐scFv, (B) Di‐scFv (EP)_8_, (C) Di‐scFv (ESP)_6_, (D) Di‐scFv (ESEP)_4_, and (E) Tri‐scFv (EP)_8_. The average ratio of fluorescence at 350 and 330 nm (F350/F330) from three replicate measurements is shown as a function of temperature (T).
**Fig. S3**. Fitted Langmuir adsorption isotherms with 95% confidence limits; (A) mono‐scFv affinity resin (ρ_Ligand_ = 0.40 µmol·mL^−1^), (B) di‐scFv affinity resin (ρ_Ligand_ = 0.08 µmol·mL^−1^), (C) di‐scFv affinity resin (ρ_Ligand_ = 0.24 µmol·mL^−1^), and (D) tri‐scFv affinity resin (ρ_Ligand_ = 0.16 µmol·mL^−1^).
