# Supplementary material for: Multimeric fusion single‐chain variable fragments as potential novel high‐capacity ligands
Source: FEBS Open Bio. 2020 Mar 3;10(4):507–14. doi: 10.1002/2211-5463.12789 (PMC7137789; doi:10.1002/2211-5463.12789)
Supplement: Supplementary file 1 — Table S1 . Temperature of mid‐point of heat denaturation (T m) of mono‐, di‐ and tri‐scFvs with selected linkers. Fig. S1. Elution profiles from IMAC chromatography for multimeric ligands with different linker lengths. (A) di‐scFv ligands with (EP)6 and (EP)8, (B) di‐scFv ligands with (ESP)4 and (ESP)6, (C) di‐scFv ligands with (ESEP)3 and (ESEP)4, and (D) tri‐scFv ligand with (EP)8. Application corresponds to 0.5 L of HEK293 supernatant. Fig. S2. Unfolding curves from nano differential scanning fluorimetry; (A) mono‐scFv, (B) Di‐scFv (EP)8, (C) Di‐scFv (ESP)6, (D) Di‐scFv (ESEP)4, and (E) Tri‐scFv (EP)8. The average ratio of fluorescence at 350 and 330 nm (F350/F330) from three replicate measurements is shown as a function of temperature (T). Fig. S3. Fitted Langmuir adsorption isotherms with 95% confidence limits; (A) mono‐scFv affinity resin (ρLigand = 0.40 µmol·mL−1), (B) di‐scFv affinity resin (ρLigand = 0.08 µmol·mL−1), (C) di‐scFv affinity resin (ρLigand = 0.24 µmol·mL−1), and (D) tri‐scFv affinity resin (ρLigand = 0.16 µmol·mL−1). [file FEB4-10-507-s001.docx]

**Multimeric fusion single-chain variable fragments as potential novel high-capacity ligands**

**Supporting Information**

Laila I. Sakhnini^1,2^*, Anja K. Pedersen^3^, Maria B. Dainiak^1^, Leif Bülow^2^

^1^Global Research Technologies, Novo Nordisk A/S, Copenhagen, Denmark

^2^Department of Pure and Applied Biochemistry, Lund University, Lund, Sweden

^3^Chemistry, Manufacturing and Control, Novo Nordisk A/S, Copenhagen, Denmark

***Corresponding author:**

L.I. Sakhnini, Division of Pure and Applied Biochemistry, Lund University, Sweden

Email: [Laila.I.Sakhnini@gmail.com](mailto:Laila.I.Sakhnini@gmail.come)

**Supporting Information Table of Contents**

**Description** **Page**

Table S1: Thermal unfolding data S2

Figure S1: Overlay of IMAC chromatograms S3

Figure S2: Unfolding curves S4

Figure S3: Adsorption isotherms S5

**Table S1.** Temperature of mid-point of heat denaturation (T_m_) of mono-, di- and tri-scFvs with selected linkers.

| **#** | **Ligand** | **T_m_**  [°C] |
| --- | --- | --- |
| 1 | Mono-scFv | 52.9 ± 0.0 |
| 2 | Di-scFv (EP)_8_ | 54.6 ± 0.1 |
| 3 | Di-scFv (ESP)_6_ | 54.3 ± 0.0 |
| 4 | Di-scFv (ESEP)_4_ | 54.2 ± 0.0 |
| 5 | Tri-scFv (EP)_8_ | 52.8 ± 0.1 |
| Data is presented as mean ± σ (n = 3). | | |


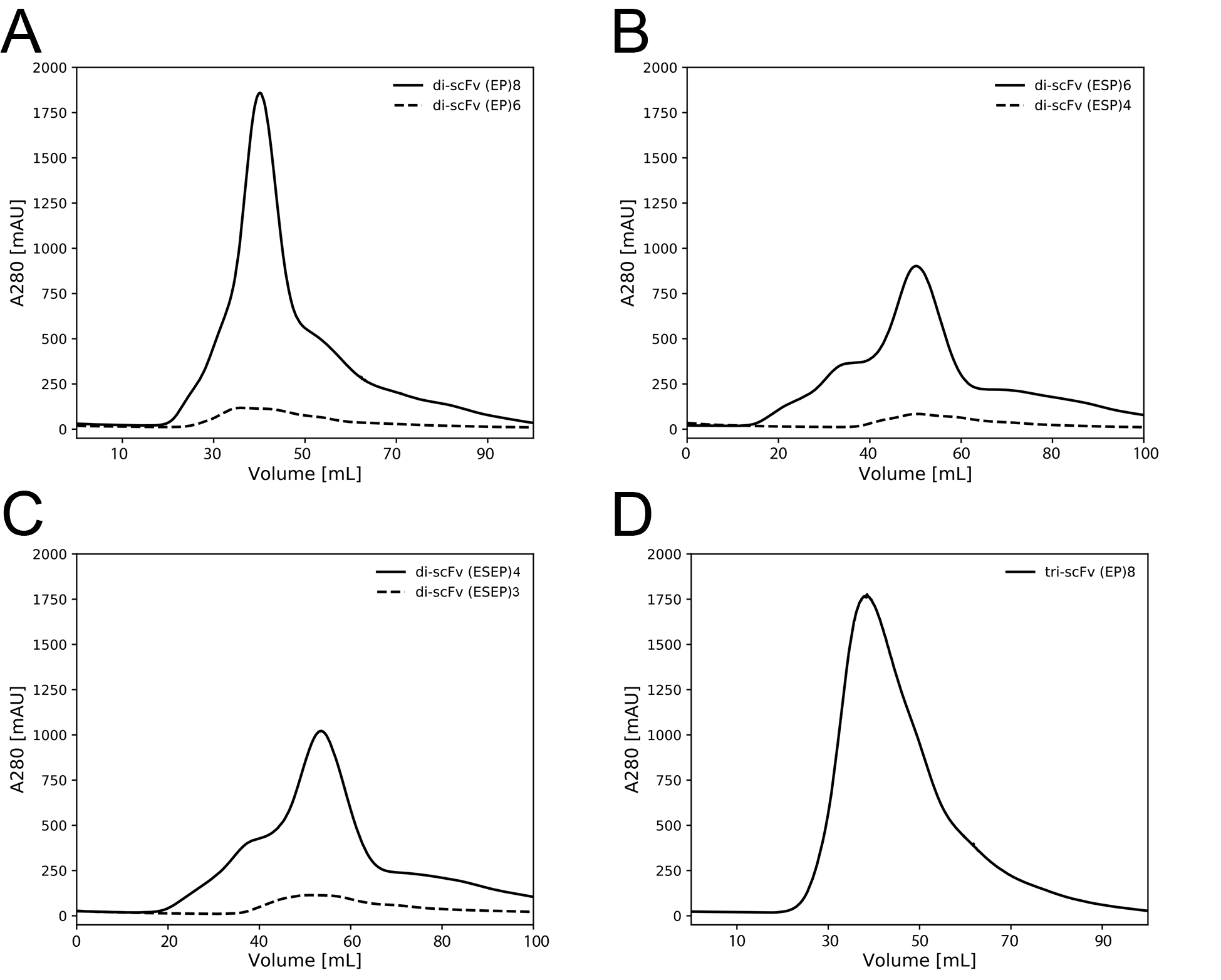


**Figure S1.** Elution profiles from IMAC chromatography for multimeric ligands with different linker lengths. (A) di-scFv ligands with (EP)_6_ and (EP)_8_, (B) di-scFv ligands with (ESP)_4_ and (ESP)_6_, (C) di-scFv ligands with (ESEP)_3_ and (ESEP)_4_, and (D) tri-scFv ligand with (EP)_8_. Application corresponds to 0.5 L of HEK293 supernatant.


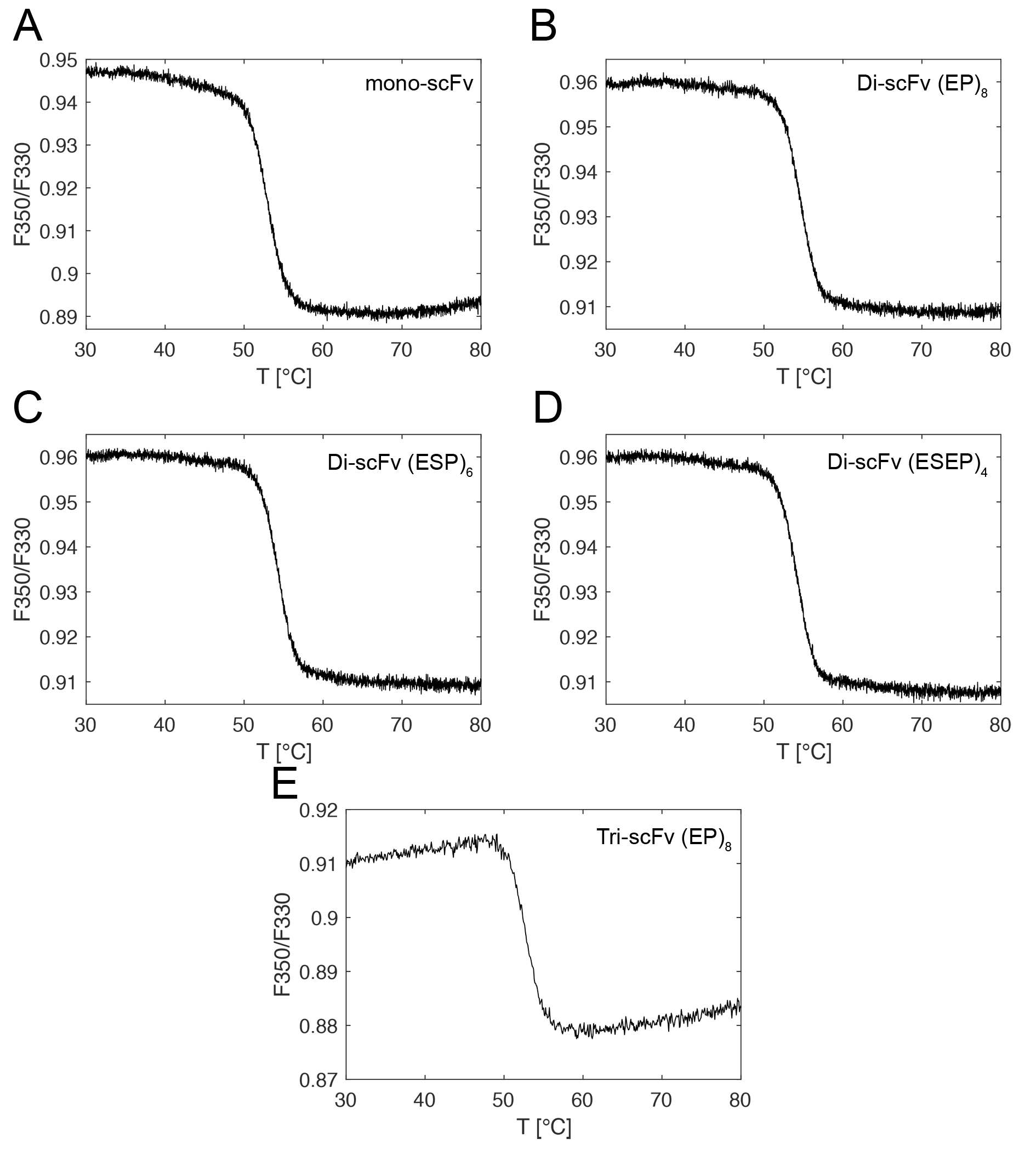


**Figure S2.** Unfolding curves from nano differential scanning fluorimetry; (A) mono-scFv, (B) Di-scFv (EP)_8_, (C) Di-scFv (ESP)_6_, (D) Di-scFv (ESEP)_4_, and (E) Tri-scFv (EP)_8_. The average ratio of fluorescence at 350 and 330 nm (F350/F330) from three replicate measurements is shown as a function of temperature (T).

**
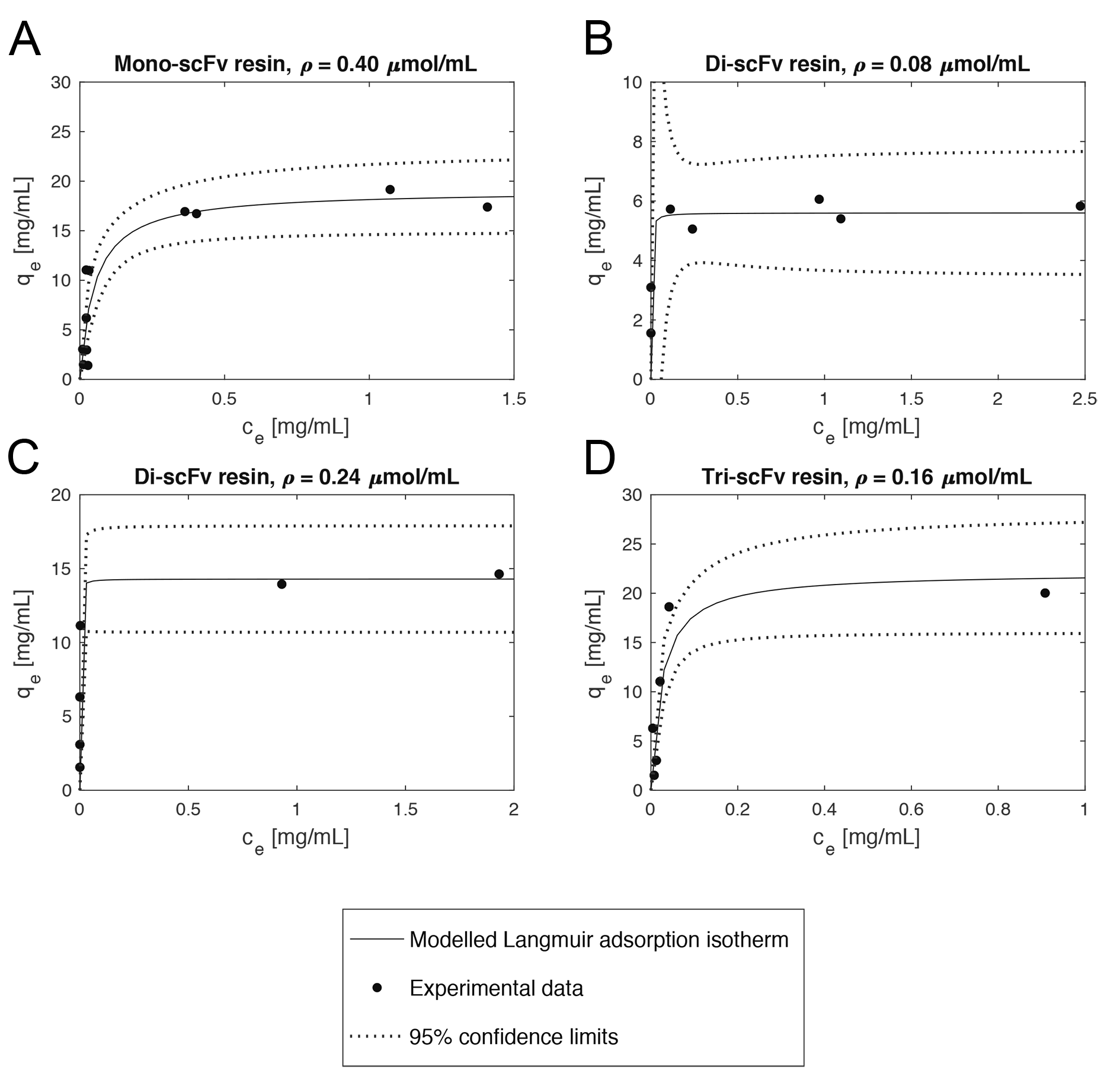
**

**Figure S3.** Fitted Langmuir adsorption isotherms with 95 % confidence limits; (A) mono-scFv affinity resin (ρ_Ligand_ = 0.40 µmol/mL), (B) di-scFv affinity resin (ρ_Ligand_ = 0.08 µmol/mL), (C) di-scFv affinity resin (ρ_Ligand_ = 0.24 µmol/mL), and (D) tri-scFv affinity resin (ρ_Ligand_ = 0.16 µmol/mL).
